# Evaluation of spiritual well-being using purpose in life (PIL) assessment among older people living in the community of South Korea: a cross-sectional study

**DOI:** 10.1186/s41182-025-00838-x

**Published:** 2025-11-28

**Authors:** Mengyi Chen, Ishtiaq Ahmad, Hira Taimur, Yoshihisa Shirayama, Miyoko Okamoto, Eun Woo Nam, Motoyuki Yuasa

**Affiliations:** 1 Department of Global Health Research, Graduate School of Medicine, Juntendo University, Tokyo, Japan; 2 Faculty of International Liberal Arts, Juntendo University, Tokyo, Japan; 3 Department of Health Administration, Yonsei University Graduate School, Wonju, Republic of South Korea

**Keywords:** Spiritual health, Purpose in life, Older adults, Ageing, Korea

## Abstract

**Introduction:**

South Korea is experiencing an ageing population, coupled with a high prevalence of mental health issues among its older people. This study aimed to identify the variables that influence spiritual health (SH), seeking to provide a stronger theoretical foundation and practical guidance for designing interventions to improve purpose in life (PIL), ultimately improving spiritual health and overall quality of life in older adults.

**Methods:**

This cross-sectional survey included 270 older adults (aged ≥ 65 years) from Wonju, Gangwon Province, and Yeoju, Gyeonggi Province, South Korea. The paper-based survey questionnaire included questions on demographic characteristics, health perceptions, and the PIL test. Descriptive statistics, Chi-square tests, and multivariate logistic regression analyses were performed using Stata 18.

**Results:**

A total of 270 older adults (mean age = 73.8 ± 6.5 years) participated in the study, with women comprising the majority (62.6%). Among the participants, 79.6% (*n* = 215) reported low levels of purpose in life (PIL), while 20.4% (*n* = 55) reported moderate levels; no participants were classified as having high PIL. Educational attainment was significantly associated with PIL, as individuals with a university degree (*p* = 0.04) or graduate-level education (*p* = 0.01) were less likely to report moderate PIL compared to those with primary or junior high school education. In contrast, living with a care recipient was strongly and positively associated with moderate PIL (*p* = 0.001). Similarly, participants who reported strong religious faith demonstrated higher odds of moderate PIL (*p* < 0.01), whereas adherence to Shintoism was negatively associated with PIL (*p* = 0.03). Interestingly, engagement in volunteer activities was also inversely associated with PIL (*p* = 0.01).

**Conclusion:**

The findings of this study suggest that PIL in older adults is shaped by a complex interplay between cultural, historical, and social factors. To address these issues, it is important to promote structured volunteer opportunities tailored to the preferences of older adults, strengthen caregiver support systems, and implement interventions that focus on alleviating social and economic difficulties. By doing so, it is expected that the sense of purpose in life among older adults will be enhanced, leading to an improvement in their overall well-being.

## Introduction

Global ageing is a significant demographic trend, characterized by increased longevity and declining fertility. This trend is profoundly changing the age structure of the world's population and posing new challenges to social, economic, and health systems. By 2030, one in six people worldwide will be 60 years or older, increasing to 2.1 billion by 2050 [1]. Much of the increase in population ageing is due to significant advances in healthcare, nutrition, and hygiene, which have contributed to an increase in life expectancy. For example, in Japan, the average life expectancy has surpassed 84 years (for women), making Japan one of the longest living countries in the world [2]. As life expectancy continues to increase, it is not only the length of life that is a goal, but also the quality of life. In an age when people can live up to 100 years, it is vital to ensure that they can live longer while remaining physically and mentally healthy and leading vibrant lives. This reflects the World Health Organization (WHO) definition of “healthy ageing”, which emphasizes maintaining functional ability and physical and mental well-being through active lifestyles and supportive environments to enable older adults to live fulfilling and dignified lives [3].

'South Korea's older adult population will exceed 10 million in 2024', states that Korea is currently facing a rapid ageing process. By 2024, approximately 20% of the total population will be 65 years and older. As with globalization, Korea's high life expectancy and low birth rate (one of the lowest in the world) are the main drivers of this trend. As the first generation of baby boomers (born between 1955 and 1963) reaches the age of 65 in 2020, the ageing trend will continue to accelerate, and it is projected that by 2025, more than 20% of the total population will be 65 years old or older, putting Korea on the path to becoming a 'super-ageing society' [4]. By 2033, the second generation of baby boomers (born between 1968 and 1974) will also reach the age of 65, further fuelling this process. Unlike older generations, these two generations are better educated and financially better off, resulting in a higher demand for quality social and healthcare services, as well as a growing demand for long-term care (LTC) services[1]. This makes the concept of healthy ageing particularly important in Korea, not only to help meet the challenges posed by an ageing population, but also to help improve the overall quality of life of older people.

The WHO defines health as a dynamic state in four dimensions: physical, mental, social, and spiritual [5]. Spiritual health (SH) refers not only to religious beliefs, but also to the ability to find meaning and purpose in life (PIL), the quest for inner growth, and a deep connection between individuals and the environment [6]. It involves the ability to cultivate positive emotions such as love and gratitude, and plays an important role in coping with life's stresses, helping people to gain a sense of inner peace and meaning in their lives, thereby enhancing their overall quality of life and well-being. For older people in particular, SH can significantly enhance their life satisfaction and help them better cope with the changes and challenges of life stages. Globally, there has been a growing emphasis on SH of older people in the pursuit of longevity while helping them achieve physical and mental balance and peace of mind [6].

While spiritual health is a multidimensional concept, purpose in life is its core component common to all definitions found in the literature [7]. Since most instruments to determine spiritual health involve questions regarding God, we consider using “Purpose in Life (PIL)” developed by Crumbaugh and Maholick most appropriate for a demographic where atheism is predominant. Purpose or meaning in life helps people stay motivated in the face of challenges and is assessed by measuring an individual's perception and experience of ‘meaning in life’ or ‘a sense of purpose in life’ [8]. SH supports individuals in finding meaning and purpose by helping them achieve a sense of inner peace and fulfilment in their lives, thus increasing resilience and well-being in the face of challenges [6, 9]. This scale is based on Viktor Frankl’s theory of meaning therapy [10, 11].

Several sociodemographic factors have been identified as significant determinants of PIL, such as gender, marital status, education level, and income [12–15]. Beyond the demographic factors, social factors also affect PIL. These encompass social relationships, participation, and family dynamics, all of which foster a sense of belonging and emotional well-being. These factors encourage individuals to engage in meaningful activities and find purpose, particularly later in life[11, 16, 17]. For older adults, family relationships serve as primary sources of support, helping them maintain their sense of purpose through caregiving roles and social connections[8, 11]. Furthermore, volunteering is recognized for its multidimensional benefits, including psychological well-being, cognitive stimulation, and identity formation [18–20]. Such activities help older adults maintain PIL and improve their overall quality of life.

On the contrary, factors such as living alone, financial stress, losing a spouse, impaired cognitive function, and mental health disorders such as depression are associated with a diminished sense of purpose [21–23]. These factors not only undermine psychological well-being, but are also linked to adverse physiological outcomes, including weakened immune function. Emerging evidence further suggests potential links between spirituality, religiosity, and PIL, as well as the role of social support systems and community resources in improving one’s sense of purpose [22–37]. However, many of these relationships remain underexplored, necessitating further empirical research to clarify their mechanisms and cultural variations.

Despite the critical importance of SH and PIL, research in South Korea has predominantly focused on older adults living in institutions or those with serious illnesses, such as advanced cancer or depression, leaving relatively few studies on healthy, independent older adults. This gap limits our understanding of the mental health and spiritual well-being of independent older adults. This study explored the variables influencing SH, aimed at establishing a more robust theoretical foundation and offering practical guidance for designing interventions that improve PIL, thus improving the spiritual health and overall quality of life of the older adult population.

## Materials and methods

### Study setting and participants

This cross-sectional study was conducted with a sample size of 300 older individuals, comprising both men and women 65 years and older. The participants were residents of Wonju, Gangwon Province, and Yeoju, Gyeonggi Province. Data collection involved administering questionnaires at daycare centers and community centers, with participants recruited through convenience sampling. The study did not employ complex sampling strategies such as stratification or clustering. The sample size was determined using G*Power 3.1, based on two-tailed test assuming a power of 0.9, alpha error probability of 0.05, and medium effect size (*ρ* = 0.3) [38].

**Fig. 1:**
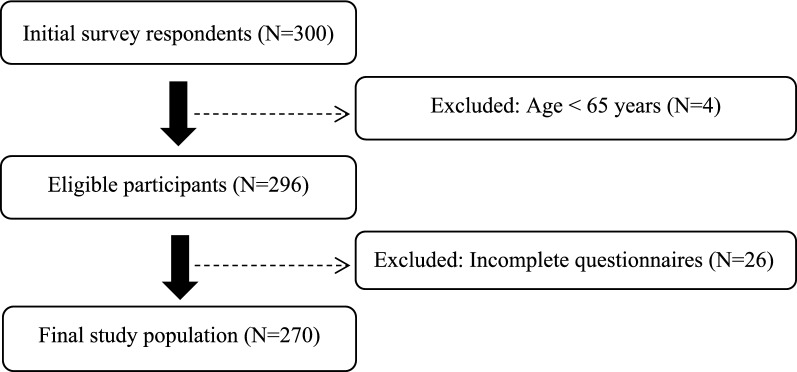
Flowchart of participant selection and exclusions

Of the survey respondents (*n* = 300), the age of the participants who did not reach 65 years (*n* = 4) and those who did not complete the questionnaire in full (*n* = 26) were excluded from the study. The final study population consisted of 270 participants (Fig. 1). Participants excluded from the analysis were those who did not meet the inclusion criteria.

### Eligibility criteria

Participants were older individuals living in the community, 65 years and older, who used facilities such as day care and community centers. All participants had stable physical and mental health conditions, lived in South Korea, and spoke Korean as their native language.

The exclusion criteria, as self-reported by the participants, included institutionalized individuals, those with a history of dementia, and those deemed unsuitable for the study by the principal investigator. Although some participants attended day care centers, all were community-dwelling individuals living independently, and the facilities were primarily used for social, recreational, and health promotion purposes, rather than long-term care or assisted living.

### Study instrument

#### Purpose in life test (PIL)

PIL scale was developed by Crumbaugh and Maholic based on Frankl’s theory in the English language in 1964, and since then it has been translated into many languages. We used the authentic Korean translation developed by Namgung in 1981 [39] as the validated Korean version of this scale in our study, as has been used in previous studies [40]. It consists of a 20-item, 7-point Likert scale. The total score ranges from 20 to 140. We applied the cutoff values recommended by the test manual authors, categorizing scores from 20 to 91 as indicating a “low sense of purpose” due to a lack of clear meaning, scores between 92 and 112 as representing a “moderate sense of purpose” within an indecisive range, and scores of 113 and above as reflecting a “high sense of purpose” or definite purpose [27]. These cutoffs were adopted in several studies, highlighting their scientific credibility [11]. The scale has demonstrated strong reliability, with split-half reliability ranging from 0.77 to 0.85, and test–retest reliability between 0.68 and 0.83. The internal consistency, measured by alpha coefficients, ranges from 0.86 to 0.9, indicating a high level of reliability and validity across diverse populations.

#### Health perception

Self-rated health and perception of health compared to the previous year.

#### Demographic characteristics

Age, sex, educational level, marital status, family status, living status, work history, income, pension, faith and religious affiliation, specific religious beliefs, social activities, and volunteer activities.

### Statistical analysis

Descriptive statistics were used to summarize demographic characteristics. To explore factors associated with the dependent variable, Chi-square tests and univariate logistic regression analyses were conducted, using a significance threshold of *p* < 0.05. Variables found to be significant in these analyses, along with covariates identified in prior research as theoretically relevant (e.g., age, sex, marital status, educational level, income), were included in a multivariable logistic regression model.

The dependent variable was the level of PIL, measured using the PIL scale and dichotomized into low and moderate levels. This categorization was used because none of the participants scored in the high range on the scale. Independent variables included age, sex, marital status, educational level, income, presence of children, currently living with children, living with someone needing care, faith or religious beliefs, Shinto beliefs, participation in volunteer activities, and self-rated health. Results are presented as odds ratios (ORs) with 95% confidence intervals (CIs).

Given the exploratory nature of this study, the multivariable logistic regression model was used to identify patterns of association rather than to estimate causal effects. We recognize that, when multiple variables are entered simultaneously, individual coefficients may reflect complex interdependencies rather than isolated effects. Therefore, the estimated odds ratios should be interpreted as descriptive indicators of association, useful for generating hypotheses rather than drawing inferential conclusions. Our findings will thus be viewed as preliminary and should be interpreted cautiously.

All analyses were conducted using Stata 18, with statistical significance defined as a two-sided *p*-value < 0.05. CIs not crossing the null were considered statistically significant [29].

## Results

The descriptive characteristics of the 270 participants included in the analysis are presented in Table 1. All variables were complete, as cases with missing data were excluded during data cleaning. Participants (mean age 73.8 ± 6.5 years), more than half of the respondents were women (62.6%). Educational attainment varied: 26.3% had completed primary or secondary education, 35.2% had high school education, and 38.5% had higher education. Most of the respondents had a spouse or partner (74.1%). About 56.3% had children but lived separately, and about 56.8% currently lived in a house with their spouse.

**Table 1:** Demographic characteristics (descriptive statistics)

Variable		*N* = 270	Percent%
Sex			
	Male	101	37.4
	Female	169	62.6
Age	73.78 ± 6.45		
Education level			
	Primary or Junior high school	71	26.3
	High school	95	35.2
	Junior college, technical college, vocational school	34	12.6
	University	44	16.3
	Graduate school	16	5.9
	Other	10	3.7
Marital status			
	Have never been married	4	1.5
	Currently have a spouse or partner	200	74.1
	Divorced from spouse or partner	9	3.3
	Spouse or partner is dead	56	20.7
	Other	1	0.4
Currently have any children and living together or separately			
	No children	15	5.6
	With children (living together only)	37	13.7
	With children (living separately only)	152	56.3
	With children (both living together and living separately)	63	23.3
	Other	3	1.1
Who currently lives in the house with you			
	Living alone	54	15.7
	Spouse (husband or wife)	196	56.8
	Your or your spouse’s parents	2	0.6
	Children	55	15.9
	Spouse of a child	28	8.1
	Grandchildren	6	1.7
	Siblings	3	0.9
	Others	1	0.3
Currently have a job that provides income			
	No income-generating job	131	48.5
	Self-employed in agriculture, forestry, or fishing (including family employees)	42	15.6
	Self-employed commercial or industrial service (including family employees)	17	6.3
	Officer of a company or organization	6	2.2
	Full-time employees	14	5.2
	Part-time/temporary employees	57	21.1
	Other	3	1.1
What kind of job have you had the longest			
	No income-generating job	33	12.2
	Self-employed in agriculture, forestry, or fishing (including family employees)	48	17.8
	Self-employed commercial or industrial service (including family employees)	45	16.7
	Officer of a company or organization	102	37.8
	Full-time employees	22	8.2
	Part-time/temporary employees	18	6.7
	Other	2	0.7
Current financial situation			
	Have enough money in my household to live without any worries	62	23.0
	Do not have much money in my household, but I do not worry much	120	44.4
	My household does not have much money, and I am somewhat worried about my finances	78	28.9
	I am very worried about my family's finances	10	3.7
Income			
	Less than 250,000 yen	112	41.5
	250,000 yen to less than 450,000 yen	87	32.2
	450,000 yen to less than 650,000 yen	48	17.8
	650,000 yen and more	23	8.5
Pension			
	No	65	24.1
	Yes	205	75.9
Live with someone who needs your care			
	No	197	73.0
	Yes	73	27.0
Faith or religious beliefs			
	I don't know	39	14.4
	No, I don't	48	17.8
	Somewhat	112	41.5
	Very strongly	71	26.3
What kind of faith or belief do you have			
	Buddhism	72	38.3
	Shinto	80	42.6
	Christianity (including Catholic)	36	19.1
	Taoism	0	0.0
Social activities			
	None of the above activities	21	5.8
	Health and sports	191	52.5
	Hobbies	48	13.2
	Community events	36	9.9
	Improvement of living environment	28	7.7
	Production and employment	16	4.4
	Safety management	7	1.9
	Education-related and cultural awareness activities	17	4.7
Volunteer activities			
	No volunteer activity	12	4.4
	Participated in volunteer activity	258	95.6
How do you feel about own health			
	Not in good health	30	11.1
	Not very healthy	86	31.9
	Fairly healthy	109	40.4
	Very healthy	45	16.7
Compared to last year, do you think you are "as well as ever"			
	No, I am not well	104	38.5
	Can't say either	53	19.6
	Yes, I am well	113	41.9

Categorical variables are presented as frequencies (*n*) and percentages (%), and continuous variables are presented as means ± standard deviations (Mean ± SD)

Table 2 presents the distribution of participant characteristics by purpose in life (PIL) category. Among the 270 participants, 215 (79.6%) had low PIL and 55 (20.4%) had moderate PIL. None of the participants scored in the high PIL range. Significant differences in PIL were observed across several demographic and psychosocial variables. Participants with moderate PIL were more likely to have completed high school (52.7%) compared to those with low PIL (30.7%) (*p* = 0.05). Living arrangements also showed a notable association: individuals in the moderate PIL group were more likely to live with children (34.6%) than those in the low PIL group (16.7%) (*p* < 0.01). Similarly, a higher proportion of those with moderate PIL lived with someone needing care (79.1%) compared to those with low PIL (20.9%) (*p* < 0.001). Religious beliefs were also associated with PIL. Participants with moderate PIL were more likely to report very strong religious faith (43.6%) than those with low PIL (21.9%) (*p* < 0.01). Additionally, Shinto belief was less common among participants with moderate PIL (18.2%) than among those with low PIL (32.6%) (*p* = 0.04). Finally, self-rated health was significantly associated with PIL (*p* = 0.02): only 3.6% of individuals in the moderate PIL group rated their health as “not in good health”, compared to 13.0% in the low PIL group.

**Table 2:** Demographic characteristics as per PIL status

		PIL		
Variable		Low %	Moderate %	*P*
Total		215(79.6)	55(20.4)	
Sex				0.45
	Male	77.2	81.1	
	Female	22.8	18.9	
Age				0.18
	65–69	35.4	43.6	
	70–74	22.3	12.7	
	75–80	22.8	16.4	
	80 +	19.5	27.3	
Education Level				0.05
	Primary or junior high school	27.4	21.8	
	High school	30.7	52.7	
	junior college, technical college, vocational school	13.0	10.9	
	University	18.1	9.1	
	Graduate school	7.0	1.8	
	Other	3.7	3.6	
Marital status				0.69
	Have never been married	1.9	0.0	
	Currently have a spouse or partner	74.4	72.7	
	Divorced from spouse or partner	2.8	5.5	
	Spouse or partner is dead	20.5	21.8	
	Other	0.5	0.0	
Currently have any children and living together or separately				< 0.01
	No children	5.1	7.3	
	With children (living together only)	10.2	27.3	
	With children (living separately only)	59.1	45.5	
	With children (both living together and living separately)	25.1	16.4	
	Other	0.5	3.6	
Currently lives in the house with children				< 0.01
	No	83.3	65.5	
	Yes	16.7	34.6	
Income				0.54
	Less than 250,000 yen	42.8	36.4	
	250,000 yen to less than 450,000 yen	30.2	40.0	
	450,000 yen to less than 650,000 yen	18.6	14.6	
	650,000 yen and more	8.4	9.1	
Live with someone who needs your care				< 0.001
	No	50.9	49.1	
	Yes	20.9	79.1	
Faith or religious beliefs				< 0.01
	I don't know	14.4	14.6	
	No, I don't	17.2	20.0	
	Somewhat	46.5	21.8	
	Very strongly	21.9	43.6	
Shinto belief				0.04
	No	67.4	81.8	
	Yes	32.6	18.2	
Volunteer activities				0.06
	No volunteer activity	3.3	96.7	
	Participated in volunteer activity	9.1	90.9	
How do you feel about own health				0.02
	Not in good health	13.0	3.6	
	Not very healthy	33.5	25.5	
	Fairly healthy	35.8	58.2	
	Very healthy	17.7	12.7	

Categorical variables were analyzed using Chi-square tests

Univariate logistic regression analysis revealed several significant associations between individual variables and purpose in life (PIL) status as shown in Table 3. Participants with a high school education had significantly higher odds of reporting moderate PIL compared to those with primary or junior high school education (OR = 2.16, 95% CI 1.01–4.61, *p* = 0.05). Living arrangements showed a notable association. Individuals whose children lived separately (OR = 0.29, 95% CI 0.13–0.63, *p* < 0.01), or both together and separately (OR = 0.24, 95% CI 0.09–0.64, *p* < 0.01), were significantly less likely to report moderate PIL compared to those living only with children. In contrast, participants who lived with children had greater odds of moderate PIL (OR = 2.62, 95% CI 1.35–5.08, *p* < 0.01). Similarly, living with someone who required care was strongly associated with higher odds of moderate PIL (OR = 3.92, 95% CI 2.10–7.30, *p* < 0.001). Religious belief also appeared to play a role. Participants without religious faith had significantly lower odds of reporting moderate PIL compared to those who were unsure (OR = 0.24, 95% CI 0.11–0.51, *p* < 0.001). In addition, individuals who adhered to Shinto beliefs had lower odds of moderate PIL than both non-religious participants (OR = 0.46, 95% CI 0.22–0.97, *p* = 0.04) and those affiliated with other religions (OR = 0.45, 95% CI 0.20–0.99, *p* = 0.05), suggesting a consistent pattern. Finally, participants who rated their health as “fairly healthy” had significantly higher odds of reporting moderate PIL than those who considered their health poor (OR = 4.43, 95% CI 1.31–25.88, *p* = 0.02).

**Table 3:** Results of univariate logistic regression analyses

Variable		Odds ratio	95%CI	*P*
Sex	Male	(Reference)	–	–
	Female	0.79	0.43–1.45	0.45
Age		1.01	0.96–1.05	0.81
Education level	Primary or Junior high school	(Reference)	–	–
	High school	2.16	1.01–4.61*	0.05
	Junior college, technical college, vocational school	1.05	0.36–3.10	0.92
	University	0.63	0.21–1.93	0.42
	Graduate school	0.33	0.04–2.72	0.30
	Others	1.23	0.23–6.52	0.81
Marital status	Have never been married	(Reference)	–	–
	Currently have a spouse or partner	0.92	0.44	0.81
	Divorced from spouse or partner	1.83	1.9	0.44
	Spouse or partner is dead	–	–	–
	Other	–	–	–
Currently have any children and living together or separately	With children (living together only)	(Reference)	–	–
	With children (living separately only)	0.29	0.13–0.63**	< 0.01
	With children (both living together and living separately)	0.24	0.09–0.64**	< 0.01
	No children	0.53	0.14–1.99	0.35
	Other	2.93	0.24–35.33	0.40
Currently lives in the house with children	No	(Reference)	–	–
	Yes	2.62	1.35–5.08**	< 0.01
Income	Less than 250,000 yen	(Reference)	–	–
	250,000 yen to less than 450,000 yen	1.56	0.79–3.08	0.20
	450,000 yen to less than 650,000 yen	0.92	0.37–2.26	0.86
	650,000 yen and more	1.28	0.42–3.85	0.66
Live with someone who needs your care	No	(Reference)	–	–
	Yes	3.92	2.10–7.30***	< 0.001
Faith or religious beliefs	I don't know	(Reference)	–	–
	No, I don't	0.24	0.11–0.51***	< 0.001
	Somewhat	0.58	0.25–1.34	0.20
	Very strongly	0.51	0.20–1.27	0.15
Shinto belief (vs. non-Shinto)	No	(Reference)	–	–
	Yes	0.46	0.22–0.97*	0.04
Shinto belief (vs. other religions)	No	(Reference)	–	–
	Yes	0.45	0.20–0.99*	0.05
Volunteer activities	No volunteer activity	(Reference)	–	–
	Participated in volunteer activity	0.34	0.10–1.10	0.07
How do you feel about own health	Not in good health	(Reference)	–	–
	Not very healthy	2.15	0.58–12.76	0.20
	Fairly healthy	4.43	1.31–25.88*	0.02
	Very healthy	2.17	0.50–13.37	0.26

1.* *p* < 0.05, ** *p* < 0.01, *** *p* < 0.001

2.Shinto belief (vs. non-Shinto): comparison between individuals with Shinto belief and those without Shinto belief (including non-religious individuals and followers of other religions)

3.Shinto belief (vs. other religions): comparison between individuals with Shinto belief and those following other religions. This analysis is restricted to participants reporting a religious belief rated as 1 (“very strongly”) or 2 (“somewhat strongly”)

4.Values were rounded to two decimal places. For example, *p* = 0.05 corresponds to an actual value of *p* = 0.047. Education level includes ‘high school’ as a category. In the Shinto belief (vs. other religions) analysis, *p* = 0.048 for participants who selected ‘Yes’

The multivariate logistic regression analysis identified several significant correlates of PIL levels, including education level, presence of children, living with someone needing care, faith or religious beliefs, Shinto beliefs, and participation in volunteer activities. Variables such as sex, age, marital status, living with children, income, and self-rated health were not statistically significant (*p* > 0.05) and did not meaningfully contribute to the model. Notably, participants with university-level or higher education had significantly lower odds of moderate PIL compared to those with primary or junior high education (university: OR = 0.21, 95% CI 0.05–0.95, *p* = 0.04; graduate school: OR = 0.03, 95% CI 0.00–0.44, *p* = 0.01). Living with someone who required care was strongly positively associated with moderate PIL (OR = 5.64, 95% CI 2.12–14.99, *p* = 0.001), suggesting caregiving may enhance life purpose. Similarly, having very strong faith was positively associated with PIL (OR = 11.26, 95% CI 2.27–55.70, *p* < 0.01). Conversely, adherence to Shinto beliefs was negatively associated with PIL (OR = 0.26, 95% CI 0.08–0.86, *p* = 0.03), consistent with the hypothesized cultural stigma. Unexpectedly, participation in volunteer activities was also negatively associated with PIL (OR = 0.13, 95% CI 0.03–0.66, *p* = 0.01), which may reflect contextual factors related to volunteer experiences.

The ‘Other’ category in living arrangements showed an extremely large odds ratio (OR = 93.65, 95% CI 2.52–3478.65, *p* = 0.01), indicating considerable instability likely due to sparse data and a small sample size within this group. The wide confidence interval underscores the need for cautious interpretation of this finding. While category collapsing was considered, the distinct nature of this group led us to retain it as is. Future studies with larger and more balanced samples are necessary to obtain more stable estimates and better understand the relationship between living arrangements and PIL.

## Discussion

This study identified several sociodemographic and psychosocial factors significantly associated with purpose in life (PIL) among older adults in South Korea. Among the participants, 79.6% exhibited low PIL levels, while 20.4% demonstrated moderate levels; notably, no participants scored in the high PIL category. Multivariate logistic regression analysis revealed key correlates of PIL, including educational attainment, caregiving responsibilities, religious beliefs, and involvement in volunteer activities.

Interestingly, in contrast to findings from Western contexts, where higher educational attainment is generally linked to a stronger sense of purpose in life (PIL) [29], our results indicate an inverse association between higher education and PIL among older adults in South Korea. It is important to note that this relationship reflects correlation rather than causation, given the cross-sectional design of the study. Several factors may help explain this pattern. One possible interpretation is that highly educated individuals, who have often experienced intense societal expectations and career pressures, may derive much of their meaning from professional roles that diminish after retirement, leading to a reduced sense of purpose. Alternatively, this finding could reflect unmeasured confounders such as post-retirement adjustment, perceived role loss, or socio-economic expectations that accompany higher education. Future longitudinal studies are needed to clarify these pathways and determine whether the observed relationship persists across different life stages and cultural contexts.

Previous research has found that living with care recipients is positively associated with PIL [30]. Consistent with these findings, our multivariate analysis (Table 4) showed a strong positive association between living with someone who needs care and PIL, suggesting that caregiving responsibilities can enhance individuals’ sense of purpose. This supports the idea that caregiver roles universally contribute to a stronger life purpose, particularly in cultural contexts like South Korea where caregiving is deeply valued as a meaningful contribution to family and community. The positive association likely reflects these cultural values and the perception of caregiving as a purposeful and respected role. Nonetheless, caregiving can also bring significant stress and risk of burnout, highlighting the critical need for supportive interventions, such as respite care and stronger social support networks, to help caregivers sustain their sense of purpose and well-being.

**Table 4:** Results of multivariate logistic regression analyses

Variable		Odds ratio	95%CI	*P*
Sex	Male	(Reference)	–	–
	Female	0.55	0.22–1.37	0.20
Age		1	0.94–1.07	0.94
Education level	Primary or Junior high school	(Reference)	–	–
	High school	1.49	0.52–4.29	0.46
	Junior college, technical college, vocational school	0.47	0.10–2.12	0.32
	University	0.21	0.05–0.95*	0.04
	Graduate school	0.03	0.00–0.44*	0.01
	Others	0.43	0.36–4.98	0.50
Marital status	Have never been married	(empty)	–	–
	Currently have a spouse or partner	0.31	0.15–1.69	0.27
	Divorced from spouse or partner	0.85	0.10–6.47	0.84
	Spouse or partner is dead	(Reference)	–	–
	Other	(empty)	–	–
Currently have any children and living together or separately	With children (living together only)	(Reference)	–	–
	With children (living separately only)	0.49	0.06–4.25	0.52
	With children (both living together and living separately)	0.45	0.08–2.53	0.37
	No children	0.34	0.05–2.58	0.30
	Other	93.65	2.52–3478.65*	0.01
Currently lives in the house with children	No	(Reference)	–	–
	Yes	1.30	0.35–4.77	0.69
Income	Less than 250,000 yen	(Reference)	–	–
	250,000 yen to less than 450,000 yen	1.30	0.49–3.44	0.60
	450,000 yen to less than 650,000 yen	0.28	0.06–1.29	0.10
	650,000 yen and more	3.06	0.59–15.98	0.18
Live with someone who needs your care	No	(Reference)	–	–
	Yes	5.64	2.12–14.99***	0.001
Faith or religious beliefs	I don't know	(Reference)	–	–
	No, I don't	1.27	0.31–5.30	0.74
	Somewhat	1.30	0.32–5.27	0.71
	Very strongly	11.26	2.27–55.70**	< 0.01
Shinto beliefs	No	(Reference)	–	–
	Yes	0.26	0.08–0.86*	0.03
Volunteer activities	No volunteer activity	(Reference)	–	–
	Participated in volunteer activity	0.13	0.03–0.66*	0.01
How do you feel about own health	Not in good health	(Reference)	–	–
	Not very healthy	2.94	0.50–17.41	0.23
	Fairly healthy	3.88	0.68–22.14	0.13
	Very healthy	2.16	0.30–15.44	0.45

*p < 0.05, **p < 0.01, ***p < 0.001

Faith and religious beliefs were positively associated with PIL, with individuals reporting very strong faith showing significantly higher odds of a strong sense of purpose (see Table 4). This underscores the potential role of spiritual practices and beliefs in enhancing life purpose [31]. Conversely, adherence to Shinto beliefs was negatively associated with purpose in life (PIL). While this observation may suggest the influence of historical or cultural factors, such as lingering perceptions related to Shinto practices, it should be interpreted with caution. The number of respondents identifying with Shinto beliefs was relatively small, and the study design does not allow causal inference or insight into underlying mechanisms. Therefore, this association should be viewed as a context-specific and exploratory finding, not a generalizable pattern. Future research incorporating more nuanced measures of religious identity, belief intensity, and cultural perceptions is needed to clarify this relationship and determine whether similar trends appear in other populations.

Interestingly, participation in volunteer activities over the past year was negatively associated with purpose in life (PIL) in our study (Table 4), a finding that contrasts with studies from Western contexts, where volunteering is generally linked to enhanced well-being and greater life purpose among older adults [32, 33]. However, this relationship should be interpreted with caution, as the cross-sectional design and limited measurement of volunteer activity (e.g., type, duration, and motivation) do not allow firm conclusions. It is possible that older adults with lower PIL are more likely to engage in volunteering in search of meaning, or that volunteer roles in this context may not always align with individuals’ intrinsic motivations. Additionally, the relatively small, urban, and homogenous sample may limit generalizability. In the Korean cultural context, volunteerism among older adults may sometimes be shaped by external expectations—such as religious, familial, or community pressures—rather than intrinsic motivation. If such activities are perceived as obligatory or misaligned with individual values, they may not contribute positively to one’s sense of purpose. Another possible explanation could be reverse causality, where individuals with lower PIL may be more inclined to seek out volunteer roles in an attempt to find meaning, but these efforts may not always yield the desired psychological benefits. Prior research in East Asian contexts, including China, suggests that the impact of volunteering on well-being may depend on factors such as role quality, structure, and autonomy [34]. Future research employing longitudinal and mixed-method approaches is needed to clarify these mechanisms and to better understand how cultural expectations shape the relationship between volunteering and PIL in older adults.

This study offers several notable strengths. It focuses on functionally independent older adults living in community settings, providing valuable insights into caregiving dynamics and non-institutional living arrangements. By incorporating sociocultural and historical factors unique to South Korea, the study offers a contextually grounded understanding of the correlates of purpose in life (PIL) in a rapidly ageing society. The inclusion of diverse psychosocial variables (including volunteerism, caregiving roles, and religious affiliation) helps identify potentially modifiable factors that may inform interventions to enhance psychological and spiritual well-being in later life.

However, several limitations must be acknowledged. The relatively small sample size limited statistical power and precluded subgroup analyses across key demographic or psychosocial dimensions. Sampling was confined to urban areas, potentially reducing generalizability to rural populations with distinct social dynamics. The reliance on self-reported data may have introduced bias, including recall and social desirability effects. Furthermore, the use of convenience sampling from community and day care centers may have resulted in a sample not fully representative of the broader older adult population, as those attending such centers may differ in terms of health-seeking behaviors, social support needs, or engagement levels. While the PIL scale captures a central dimension of spiritual health, it does not reflect the multidimensional nature of spirituality, possibly limiting the depth of insight into participants’ spiritual well-being. In addition, because all variables were entered into the multivariable model simultaneously, some coefficients may be influenced by inter-variable correlations, residual confounding, or overadjustment. This analytical strategy was adopted to explore patterns of association rather than to estimate causal effects. Accordingly, the reported associations should be regarded as descriptive and hypothesis-generating, and not as evidence of independent or directional effects. Future studies employing longitudinal designs and causal modeling frameworks are needed to clarify these relationships and to more precisely identify pathways linking sociocultural and psychosocial factors to purpose in life.

Building on these findings and acknowledging the study’s limitations, it is essential to consider the broader cultural and regional contexts shaping purpose in life (PIL) among older adults in South Korea. High rates of social isolation, especially among the one-fifth of older adults living alone, contribute to psychological distress and elevated risks of lonely deaths, particularly in rural areas with shrinking family networks [35]. Economic insecurity further exacerbates these challenges, as reflected by suicide rates in this population that are more than 2.7 times higher than those in Japan [36]. Historical legacies, including the introduction of Shintoism during Japanese colonial rule, may contribute to lingering stigma that partly explains the observed negative association between Shinto adherence and PIL [41, 42]. However, these interpretations remain tentative, as our study was not designed to directly assess the historical or sociopolitical factors underlying religious identity. The present findings should therefore be regarded as context-bound and exploratory, underscoring the need for future studies employing mixed-methods and cross-cultural comparisons to examine whether this association persists across different settings or is specific to the Korean context. Addressing these complex issues requires culturally sensitive interventions that strengthen caregiver support, promote social engagement, and foster inclusive spiritual practices. Future research should explore PIL across urban and rural populations, utilize longitudinal designs to clarify causality, and incorporate cross-cultural comparisons to determine whether these patterns are unique to South Korea or more broadly applicable.

## Conclusion

This study identified several factors associated with purpose in life (PIL) among older adults in urban South Korea, including education level, caregiving responsibilities, faith and religious beliefs, Shinto adherence, and volunteer participation. The unique cultural and historical context—such as the legacy of Shintoism and the rising prevalence of older adults living alone—likely contributes to social isolation and variations in PIL within this population. However, the cross-sectional design, urban-focused sampling, and reliance on self-reported measures limit causal inferences and the generalizability of findings.

Future research using longitudinal and more diverse samples is needed to clarify these relationships across different regions and over time. Nonetheless, these findings highlight the importance of culturally sensitive interventions that support meaningful volunteer opportunities, enhance caregiver support, and address social and economic challenges. Such efforts may strengthen social connections and help older adults cultivate a greater sense of purpose, contributing to healthier ageing in South Korea.

## Data Availability

All data generated or analyzed during this study are included in this article and its supplementary material files.
